# First Identification of Amphidinols from Mexican Strains and New Analogs

**DOI:** 10.3390/toxins15020163

**Published:** 2023-02-16

**Authors:** Lorena M. Durán-Riveroll, Jannik Weber, Bernd Krock

**Affiliations:** 1 CONACyT-Departamento de Biotecnología Marina, Centro de Investigación Científica y de Educación Superior de Ensenada, Ensenada 22860, Baja California, Mexico; 2 Alfred Wegener Institut-Helmholtz Zentrum für Polar und Meeresforschung, 27570 Bremerhaven, Germany; 3 Hochschule RheinMain, 65428 Rüsselsheim, Germany

**Keywords:** phycotoxin, polyketide, LC-MS/MS, benthic dinoflagellate, secondary metabolites

## Abstract

The genus *Amphidinium* has been the subject of recent attention due to the production of polyketide metabolites. Some of these compounds have shown significant bioactivities and could be related to species interactions in the natural benthic microenvironment. Among these compounds, amphidinols (AMs) are suspected to be related to fish kills and probably implicated in ciguatera symptoms associated with the occurrence of benthic harmful algal blooms (bHABs). Here, we present the first report of a variety of AMs produced by cultured strains from several species from the Mexican Pacific, the Gulf of California, and the Gulf of Mexico. Through ultra-high performance liquid chromatography coupled with tandem mass spectrometry (UHPLC-MS/MS), ten previously known AMs (AM02, -04, -05, -06, -07, -09, -11, -14, -15, and -17), four recently reported AMs (N7, N8/N9, N12, and N13), and three new variants (U1, U2, and U3) were identified. Of the twelve analyzed *Amphidinium* cultures, five were not AM producers, and the cell quotas of the remaining seven strains ranged from close to nondetectable to a maximum of 1694 fg cell^−1^, with many intermediate levels in between. The cultures from the Mexican North Pacific coast produced AMs in a higher quantity and variety than those from worldwide locations. This is the first study of AMs from Mexican *Amphidinium* strains, and our results confirm the relevance of continuing the investigation of the genus bioactive metabolites.

## 1. Introduction

Among benthic dinoflagellates, the genus *Amphidinium* is one of the most abundant worldwide [1] with 97 taxonomically accepted species to date [2], though the list is constantly being reviewed. The genus is highly diverse, and several species can be found in various habitats and temperatures, showing a diversity of trophic modes [1,3,4]. Therefore, and for the relative easiness of culturing, several species have been used as models for genetics, polyketide production, and photosynthesis research [5].

Some species of the genus have been reported as toxin producers and have been related to benthic harmful algal blooms (bHABs) in several coastal zones, such as the Italian Mediterranean Sea, the North Arabian Sea, the eastern coast of South Australia, and the coasts of Portugal [6,7,8,9]. In some of these events, *Amphidinium* bHABs have been related to fish kills, with *A. carterae*, *A. gibossum*, *A. massartii,* and *A. operculatum* reported as ichthyotoxin producers [5,6,8,9,10,11]. However, besides fish toxicity, some polyketides produced by *Amphidinium* species have been shown to possess potentially useful bioactivities, such as antifungal, cytotoxic, and antimicrobial bioactivities [12,13,14]. Yet, only some of the members of the genus or the species, and not even all members of the same strain, can synthesize these polyketides [15].

A common type of polyketides are amphidinols (AMs), which have been reported to be produced by *A. carterae* and other closely related species. About 20 known analogs possess properties that include hemolytic and antifungal activities [8]. Since the first family member was reported as a potent antifungal agent in 1991 by Satake et al. [13], amphidinols and their closely related analogs, e.g., luteophanols, lingshuiols, and carteraols, have formed an important group of natural bioactive marine products [13,16]. Because of their high structural similarities, all the above-mentioned variants are operationally included in the term “amphidinol” in this manuscript unless specifically named.

In many cases, AMs and their analogs are the causative molecules for reported biological activities. Their reported mode of action is related to increased membrane permeability due to the AM binding to the cell lipid bilayer [16]. Due to their size, it is likely that AMs have a higher number of structural variations than currently known. Hypothetically, this putative variety increases the possibility of discovering new AM variations during the analytical process [17]. No AM-related human intoxications are registered to date [18]; however, there are reports of fish kills, and some compounds produced by members of this genus have been shown to cause the lysis of bacteria and microalgae and hemolysis activity [19,20,21].

In Mexico, the genus is considered an emerging risk in coastal zones, but little research has been conducted, and there is still little molecular information available [22]. The registered species rely mostly upon morphological characterization; to our knowledge, this is the first AM analysis on Mexican *Amphidinium* strains.

The aim of the present study is the quantification and identification of AMs of 12 individual samples of the algal genus *Amphidinium* by using several UHPLC-MS/MS operation modes. This is especially relevant because the toxin profiles of many strains are still completely unknown, making it difficult to raise hypotheses about their chemical, biological, or toxicological properties. Furthermore, the large size of the AM increases the probability of the presence of different chemical variants, which has not been systematically investigated. Potentially new AM variants detected in this study will be integrated into an existing specific UHPLC-MS/MS method in the selected reaction monitoring (SRM) mode for the detection and quantification of all described AM variants.

## 2. Results

### 2.1. New Amphidinols

For the detection of potentially undescribed AMs, each individual strain was analyzed with the 14 neutral loss (NL) experiments listed in Table 1. In these measurements, a total of 15 experiments resulted in peaks with the corresponding Q1-Masses (Table 2) in 7 of the 12 strains. In addition, full-scan (FS) measurements (Table 3) were conducted and resulted in the detection of six additional Q1-Masses that were produced by four individual strains, which were not detected with the NL measurements. 

In Table 2 and Table 3, collision-induced dissociation (CID) spectra of all NL-detected Q1-Masses (Table 2) and FS measurements in the mass range between *m*/*z* 1000 and 1800 were recorded (Table 3). Three of these 20 detected Q1-Masses (*m*/*z* 1242, 1384, and 1486) were identified as AM-related compounds due to the typical AM fragmentation patterns observed in their CID spectra (Table 4).

#### 2.1.1. Variant U1

**Figure 1:**
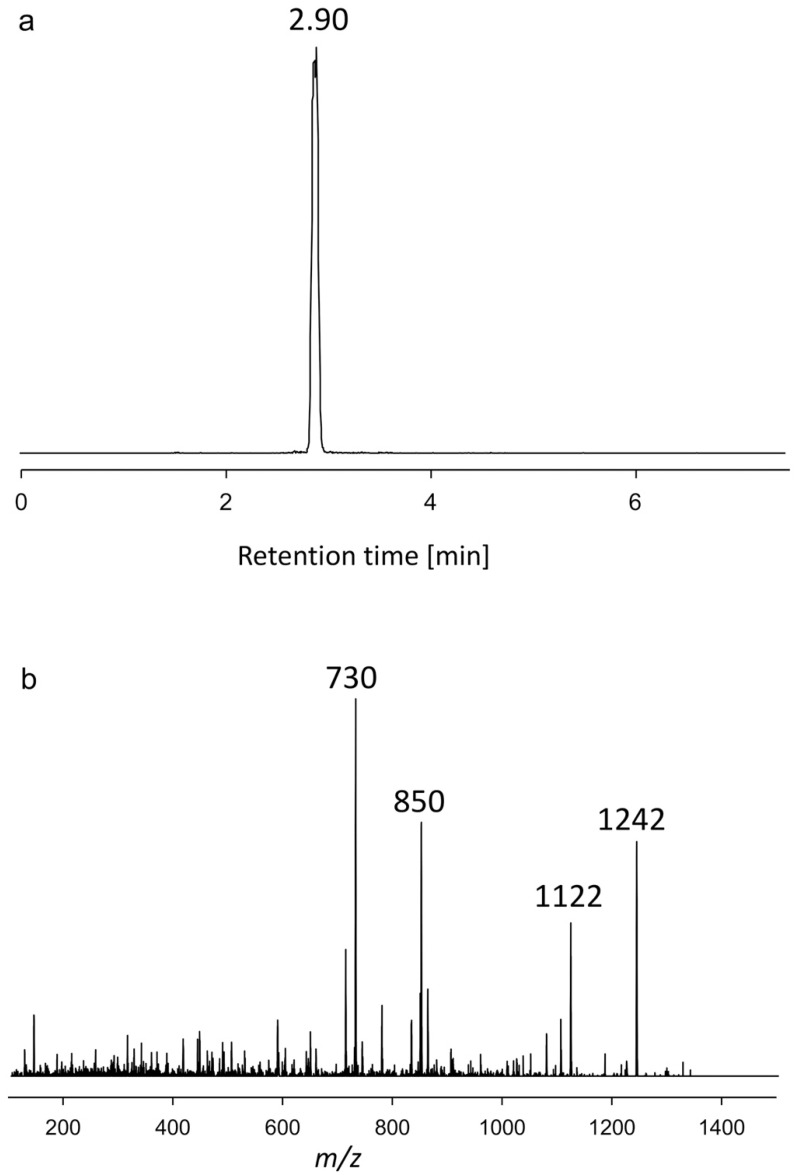
(**a**) Chromatogram showing the retention time. (**b**) Collision-induced dissociation (CID) spectrum of *m*/*z* 1242 (2.90 min) of the strain AxSQ175, named U1.

In the NL scan of 392 Da, strain AxSQ175 showed a Q1 peak at *m*/*z* 1242 and a retention time of 2.90 min. Following the NL scan for sulfated compounds, the CID spectrum of U1 showed a transition from [M+Na]^+^ *m*/*z* 1242 to the fragment *m*/*z* 1122 (Figure 1). This loss corresponds to the elimination of 120 Da, which is consistent with a loss of sodium hydrogen sulfate (NaHSO_4_), as it has been shown for AM12. The other two abundant fragments of U1 were *m*/*z* 850 and *m*/*z* 730, which also showed a mass difference of 120 Da and thus most likely resulted from the same C-1′/C-1 bond cleavage: a fragment of *m*/*z* 850 with the sulfate group and a fragment of *m*/*z* 730 without it. Both fragments have a mass difference of 392 with their precursors (*m*/*z* 1,242 and 1,122, respectively), indicating the mass of the lipophilic part of U1. A lipophilic part of the AM with a mass of 392 Da is observed in many AMs (e.g., AM2, -4, -6, -10, [19] -20, and -21 [16]), all possessing a conserved lipophilic part comprising C1′ to C22′ in the molecule. For this reason, it is assumed that this part is conserved among the above-mentioned AMs and the new variant U1. In contrast, the characteristic fragments *m*/*z* 850 and *m*/*z* 730 representing the hydrophilic part of the molecule have not been observed thus far, and accordingly present a new structural feature.

#### 2.1.2. Variant U2

**Figure 2:**
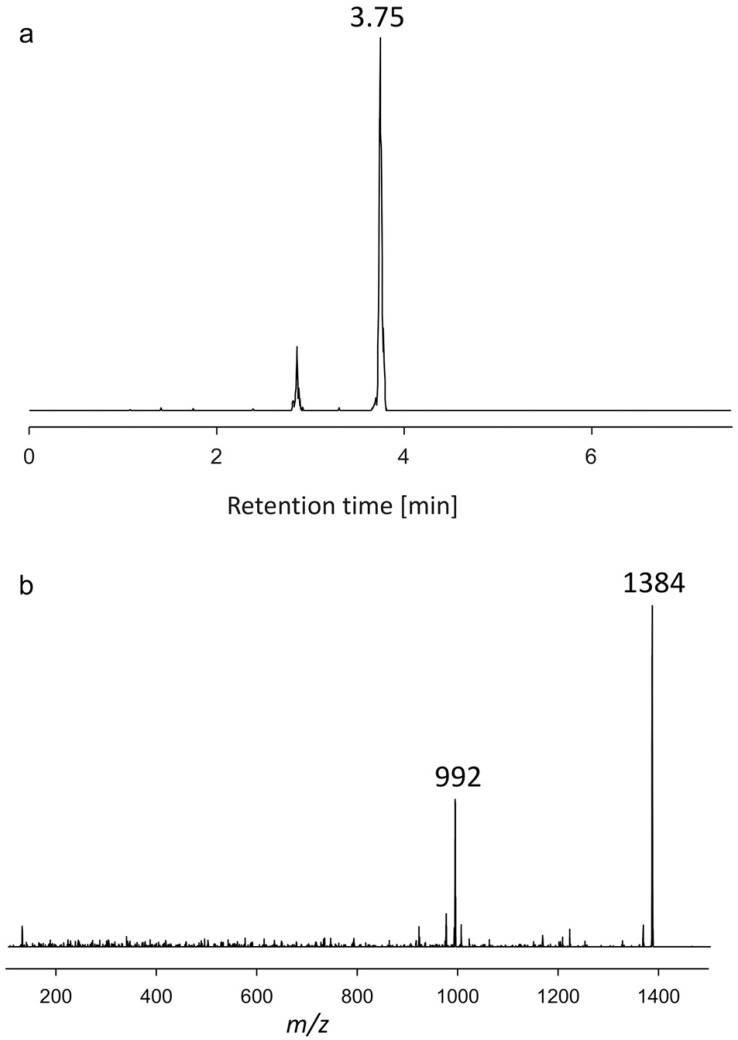
(**a**) Chromatogram showing the retention time. (**b**) Collision-induced dissociation (CID) spectrum of *m*/*z* 1384 (3.75 min) of the strain AA60, named U2.

The variant U2 showed a retention time of 3.75 min, had a mass of *m*/*z* 1384, and shared a loss of 392 Da (transition *m*/*z* 1384 > 992) resulting from the C-1′/C-1 cleavage with U1, indicating the presence of the same structural element of the lipophilic C1′-C22′ part of the molecule (Figure 2). The hydrophilic part of U2 with the mass of *m*/*z* 992 has not been reported to date, and therefore little can be said about the structural features of this part of the molecule as very few low-mass fragments were formed under CID conditions. However, despite their identical lipophilic part, U1 and U2 differ in the size of their hydrophilic part and in the fact that U1 is a sulfated AM, whereas U2 is not.

#### 2.1.3. Variant U3

**Figure 3:**
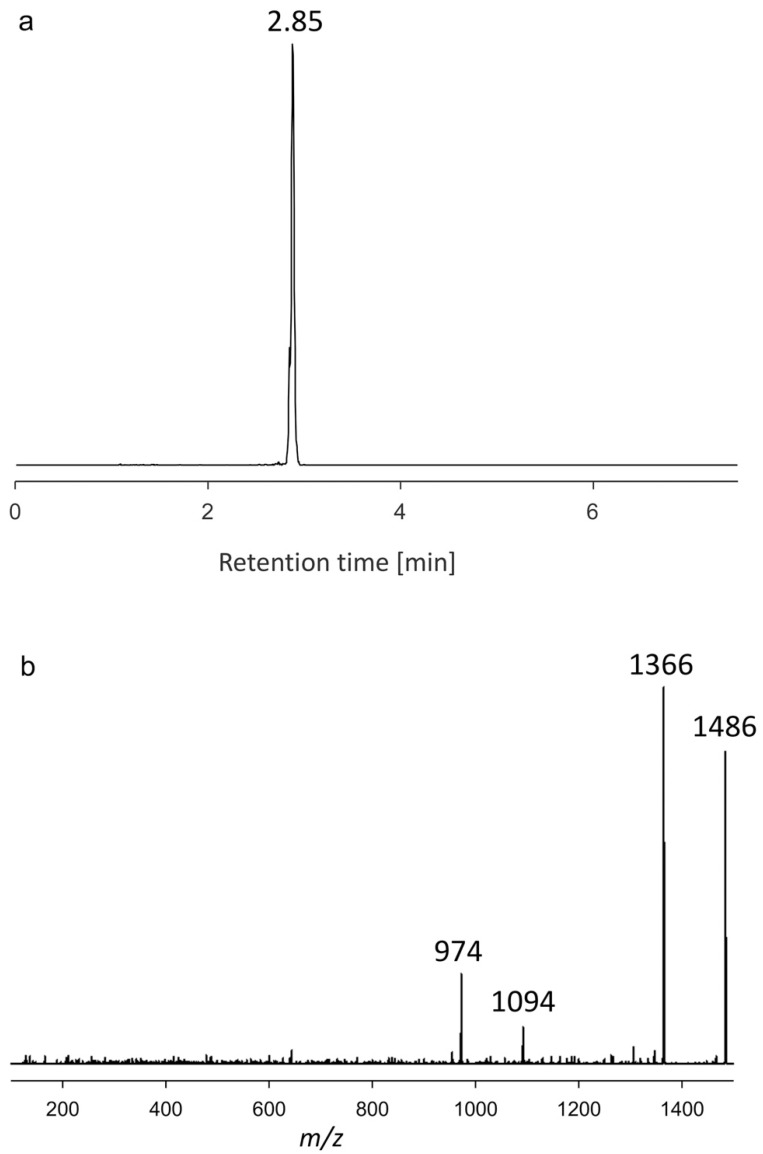
(**a**) Chromatogram showing the retention time. (**b**) Collision-induced dissociation (CID) spectrum of *m*/*z* 1486 (2.85 min) of the strain AA60, named U3.

The variant U3 showed a retention time of 2.85 min and a [M+Na]^+^ of *m*/*z* 1486. Similar to compound U1, the CID spectrum of U3 displayed four major fragments (Figure 3), which, as in the case of U1, are characterized by two pairs with a difference of 120 Da each (*m*/*z* 1486/1366 and 1094/974). As for U1, this is strong evidence of sulfation of the U3 hydrophilic arm. The elimination of the sulfate group in the form of NaHSO_4_ results in the fragment *m*/*z* 1366, whereas the C-1′/C-1 cleavage, resulting in the loss of the lipophilic arm, gives the fragment *m*/*z* 1094. This fragment can also eliminate the sulfate group resulting in *m*/*z* 974. The fact that the sulfate group can be eliminated after the elimination of the lipophilic arm is strong evidence that the sulfate group is located in the hydrophilic arm. Similar to the case of U1 and U2, the mass of the hydrophilic portion of U3 has not been reported to date, and thus these have to be regarded as novel AMs. Due to the stability of AM sodium adducts, only a few fragments are formed under CID conditions, and accordingly, little structural information can be deduced from their spectra.

### 2.2. Amphidinol Profiles 

Several already described and novel AMs were detected in seven of the 13 investigated *Amphidinium* strains (Table 5). Among the toxigenic species were *A. operculatum*, *A. massartii, A. eilatiensis*, and other isolates not yet identified at the species level. Out of the 12 strains analyzed, five have not yet been identified by molecular means. The five non-AM-producing strains were AtLPZ38, AA40, AxLT112, AxLT113, and AxRoq139. The AM detection limits of these five strains were in the range between 0.09 and 0.8 fg cell^−1^, depending on available biomass. AM cell quotas varied strongly from 1 fg cell^−1^ of a unique amphidinol, AM15, in the *A. massartii* strain AA39, to 1694 fg cell^−1^ of the total AMs (10 different variants) in the *A. eilatiensis* strain AeSQ181. This strain showed not only the highest quantity, but also the highest diversity of the produced AMs. 

**Figure 4:**
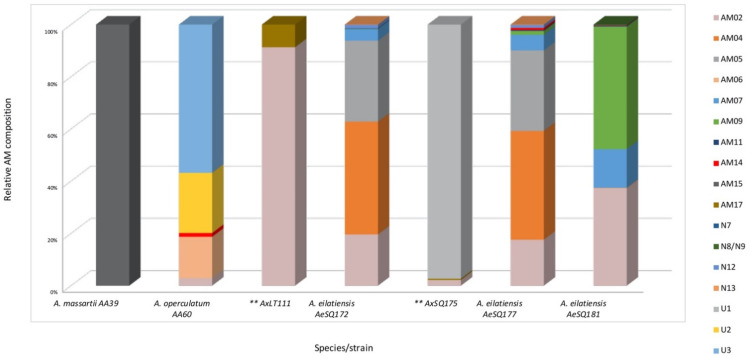
Relative AMs, estimated as LPD equivalents. ** = unidentified species. Information available in Table S1 in Supplementary Materials.

The relative AM profiles of the toxigenic strains are shown in Figure 4. Different species seem to produce different AMs. Almost every strain showed a unique profile pattern.

The profile of *Amphidinium massartii* strain AA39 consisted purely of AM15, though in a low quantity (1 fg cell^−1^), whereas the other isolate from the same species, AmLT112, was a non-AM producer. The *A. operculatum* strain AA60 mostly produced the newly discovered U3 and U2 (56.8 and 23%) and AM06 (15.8%). The strain AxLT111, an unidentified species, produced only two AMs and was almost entirely an AM02 producer, accounting for more than 90% of its relative composition. The remaining 8.7% belonged to AM17. The other unidentified strain, AxSQ175, produced almost only the newly reported variant U1 (97.3%), and AM02 and AM17 accounted for the remaining 2.7%. The *A. eilatiensis* strain profiles of AeSQ172 and AeSQ177 were quite similar, with the only difference being that the latter produced AM14, though in a low concentration (0.8%). Finally, the strain AeSQ181 of the same species was shown to be a prolific AM producer, mostly of AM09 (47%), AM02 (37.3%), and AM07 (14.8%). This strain also produced seven other AMs, which accounted for the remaining 0.9%. These results show that these strains of *A. eilatiensis* possess the most complex AM profiles, consisting of eight, nine, and eleven different AMs.

## 3. Discussion

### 3.1. Novel Amphidinols

Amphidinols are large polyketides characteristic of a marine dinoflagellate origin with molecular weights typically above 1000 Da. Their mere size allows for many modifications of the structural backbone, resulting in a potentially high number of variations. To date, about 50 AM variants have been identified and characterized at different levels. Some of their structures have been fully elucidated by nuclear magnetic resonance spectroscopy (NMR), and others only by mass spectrometric techniques and/or functional assays. Although NMR is the gold standard in natural product chemistry, it requires purified compounds in the lower mg range, which are difficult to obtain without preparative laboratory facilities, and even those laboratories have failed to correctly identify AMs in the past.

In contrast, mass spectrometry (MS) gives much less structural information than NMR, but has the significant advantage in that it can be performed in the pg range (i.e., roughly eight orders of magnitude less), does not require isolation or purification of single compounds, and can be performed on mixtures such as algal raw extracts. The high sensitivity and low sample preparation needed make MS an ideal tool for novel compound exploration. This is facilitated in the special case of AMs by two characteristics of this compound class: (1) a characteristic cleavage site between two vicinal hydroxyl groups in a highly conserved structural element of the AM (C1/C1′ cleavage), and (2) a relatively low structural variability of the lipophilic arm of the molecule that results in a small number of NLs across all AM variants, which can specifically be searched for and detected by NL scans in tandem MS.

In this work, 14 masses were detected in the analyzed *Amphidinium* strains, out of which three were confirmed as novel AMs and operationally named U1–U3. As mentioned above, the structural information of the CID spectra is limited, especially in the case of AMs, since their CID spectra display only a few fragments; however, sulfated and nonsulfated AMs can easily be distinguished by a NL of 120 Da, as this is indicative of sulfation. This was observed in U1 and U3, whereas U2 constitutes a nonsulfated new AM. This assumption was further confirmed by the chromatographic behavior of the three compounds. Compound U2 showed a retention time of 3.75 min, and the more polar sulfated compounds U1 and U3 eluted almost a minute earlier, at 2.92 min (Table 4), due to weaker retention at the lipophilic stationary phase. 

The novel AMs were detected in the middle (U2 and U3) and high (U1) fg cell^−1^ range, representing a significant fraction of the total AM profiles. In the case of the strain AxSQ175, compound U1 was the main AM that represented more than 95% of the total AM profile, indicating that the structural variability of AMs has not yet been fully explored.

### 3.2. Confirmation of Detected Amphidinols

The *Amphidinium* strains were screened with an SRM method containing transitions of known AMs [17]. Positive hits detected by SRM measurements were solely based on the defined transitions implemented in the method, and further confirmation was needed. Therefore, the CID spectra of all Q-1 Masses of the peaks of the SRM and NL scans were recorded. Under the electrospray ionization used in this work, sodium adducts of AMs were mainly formed [17], which are known to stabilize polyketides and thus lead to low fragmentation and, accordingly, CID spectra displaying a few fragments [23,24] (Figure 1, Figure 2 and Figure 3). This, in turn, allows for rapid recognition of typical fragmentation patterns corresponding to those of AMs. In this process, all transitions were found in the SRM scans (data not shown), and out of the 20 Q1-Masses found by exploratory experiments, three masses could be identified as novel AMs, which were operationally named U1–U3 as described previously. Despite identifying U1 to U3 as members of the AM family, their exact chemical structures still need to be elucidated by NMR spectroscopy in the future, as well as their toxicological effects.

### 3.3. Amphidinol Cell Quotas, Profiles, and Geographical Patterns

The AM cell quotas found in this study ranged from nondetectable to a maximum of 1694 fg cell^−1^, with many intermediate levels. Little is known about AMs’ ecological role(s), and these differences raise numerous questions. Still, the ecological function of the structurally related karlotoxins has been hypothesized to be involved in grazing, defense, and prey capture [25]. Due to their structural similarity, a comparable ecological function could be assumed. It is also known that environmental factors such as temperature and nutrient availability may influence toxin production in dinoflagellates and may partly explain different AM cell quotas across strains from other locations and species.

The strains analyzed in this work displayed, in general, very distinct AM profiles, indicating a high structural and genetic variability in the compounds and their producing organisms. Exceptions from this general observation are the *A. eilatiensis* strains AeSQ172 and AeSQ177, which were isolated from the same geographic location, belong to the same species, and show similar AM profiles (Figure 4). However, it does not seem to be a species-specific AM profile since the strain AeSQ181 was also isolated from the same location, belongs to the same species, and showed a different profile, yet includes all of the AMs displayed by the other two strains.

**Figure 5:**
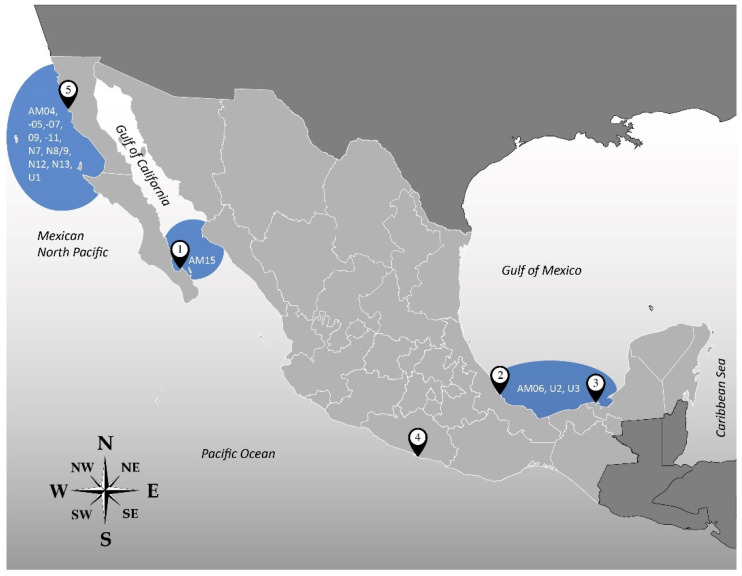
Geographical distribution of AMs. The AMs in the blue bubbles refer to the AMs detected exclusively in those locations.

There seems to be a geographical AM distribution within the *Amphidinium* strains analyzed in this study (Figure 5). Three strains were isolated from the Gulf of California, but only AA39 produced AMs, and in very low abundance. Intriguingly, the only AM produced was AM15, and this was the only producing strain. Two out of four isolates from the Gulf of Mexico did not produce any AMs. However, the remaining strains were the only ones producing AM06, U2, and U3; these were not found in any strain from another geographical region. The four strains from the Mexican North Pacific were very prolific AM producers; ten AMs (AM04, AM05, AM07, AM09, AM11, N7, N8/N9, N12, N13, and U1) were found exclusively in these strains. Finally, the only analyzed strain from the Mexican South Pacific (AxRoq139) did not produce any AMs. On the other hand, only three AMs (AM02, -14, and -17) were detected in more than one location. This distribution could be related to temperature, nutrient availability, and/or species, but further investigations are needed to unveil these puzzles. However, given the scarcity of available information on Mexican strains, this apparent geographical distribution should be taken cautiously, and further research is needed.

## 4. Conclusions

Future work is needed to structurally characterize novel AMs by NMR spectroscopy. Furthermore, to obtain insight into the ecological functions of AMs, more research is needed in the form of co-cultivation experiments with different protistan species or potential *Amphidinium* grazers, as well as with different nutrient, temperature, and light regimes. In this context, investigating if environmental stimuli can trigger AM expression may add to the knowledge on the ecological relevance of these compounds, their mitigation, or, given their biological activities and pharmacological potential, their massive production.

## 5. Materials and Methods

### 5.1. Isolation, Culture, and Harvest of Amphidinium Strains

**Figure 6:**
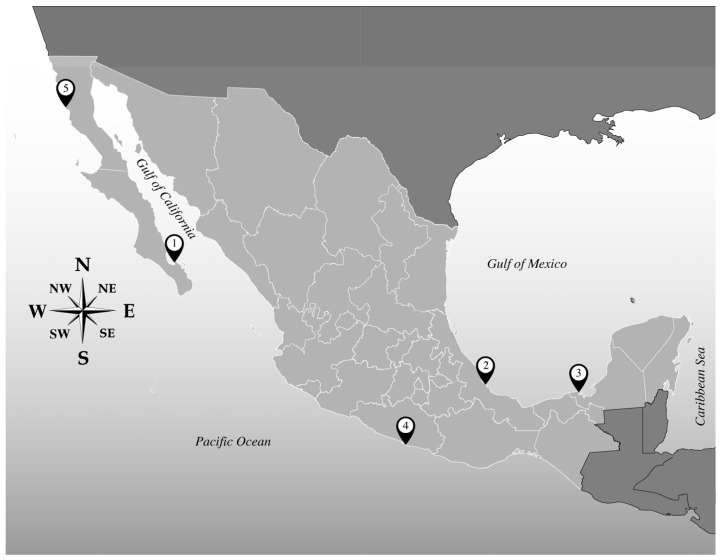
Sampling locations along Mexican coasts.

Samples for *Amphidinium* cell isolation were collected from different locations along Mexican coasts and at different times (Table 6). To establish monoclonal cultures, samples were collected from Balandra Bay, Baja California Sur, located in the southern Gulf of California (24°19′23.89″ N 110°19′44.45″ W, January 2018); the Veracruz Reef System, Veracruz (19°11′56.02″ N 96°4′5.0″ W, May 2018), and Laguna de Términos, Campeche (18°38′32.28″ N, 91°45′30.86″ W January 2018), both located in the Gulf of Mexico; Roqueta Island, Guerrero, situated in the southern Mexican Pacific (16°49′20.94″ N, 99°54′16.50″ W, June 2019); and San Quintín, Baja California (30°27′14″ N 116°00′15″ W), located in the northern Mexican Pacific (Figure 6). *Amphidinium* species were determined through morphological (light and scanning electron microscopy) and molecular analysis through the amplification of the D1 and D6 variable domains of the LSU rRNA gene (not shown), except for those designated as unidentified.

Substrates (macroalgae and seagrass) were collected by snorkeling in shallow waters, and only in the case of AxRoq139 was the sample taken using a 20 µm mesh plankton net. Live samples were transported in site water in 50 mL conical plastic centrifuge tubes (Corning, New York, NY, USA) in ice chests to maintain a temperature of around 24 °C during transportation. Once at the laboratory facilities, samples were examined under a stereo-dissecting microscope (Discovery.V8, Zeiss, Göttingen, Germany). Macroalgae and seagrasses were placed in 90 × 15 mm plastic Petri dishes and gently brushed. Single *Amphidinium* cells were isolated by being micropipetted into sterile 96-well microplates containing 200 mL of 50%-strength sterile GSe growth medium [26], modified without soil extract, and supplemented with GeO2 (final concentration of 2.5 mg L^−1^) to inhibit diatom growth. The nutrient medium was prepared with filtered autoclaved seawater at a salinity of 36. Clonal isolates were incubated at 25 °C on a 12:12 h light:dark regime and at an illumination of 50 mmol photons m^−2^ s^−1^.

Once active cell division was observed (after 7–14 days), the contents of each well were transferred into 24-well microplates with 2 mL of growth medium. After subsequent growth, every isolate was transferred into 60 × 15 mm sterile plastic Petri plates with 15 mL of full-strength modified GSe nutrient medium. Isolates are maintained at the Microalgae Chemical Ecology Laboratory in the Marine Biotechnology Department at the Ensenada Center for Scientific Research and Higher Education in Ensenada, Baja California (CICESE).

Each isolate was further grown in 125 mL Erlenmeyer flasks with 100 mL of GSe medium for 20 to 25 days under the previously described growth conditions. *Amphidinium* cells were harvested by centrifugation at 3000× *g* and 4 °C for 5 min (Heraeus Megafuge 40R, Thermo Scientific, Waltham, MA, USA) in 50 mL centrifuge tubes. Cell samples were taken for live cell counting in a Sedgwick–Rafter chamber under a light microscope (Motic-BA310E, Hong Kong, China) at 100×. The supernatant was removed, and cell pellets were transferred to 2 mL microtubes (Eppendorf, Hamburg, Germany), where they were centrifuged at 3000× *g* and 4 °C for 5 min (5415 R, Eppendorf, Hamburg, Germany). Samples were frozen at −80 °C and freeze-dried (FreeZone, Labconco, Kansas, MO, USA).

### 5.2. Amphidinol Extraction

The solid pellets were poured into cryovials (Sarstedt, Nümbrecht, Germany), and the microtubes were rinsed with 1 mL of methanol (HPLC-grade, Merck, Darmstadt, Germany), which was added to the cryovial with the sample. In addition, ca. 0.9 g of Lysing Matrix D (Thermo Savant, Illkirch, France) was added to the sample. The cells were lysed by reciprocal shaking at a speed of 4.5 m s^−1^ for 45 s in a Bio 101 FastPrep Instrument (Thermo Savant, Illkirch, France). Subsequently, the vials were centrifuged for 15 min at 16,100× *g* (5415 R, Eppendorf, Hamburg, Germany), and 500 µL of the supernatants were transferred into a 0.45 µm spin filter (Merck Millipore, Darmstadt, Germany), which was centrifuged for 30 s at 2300× *g*. The filtrates were transferred into 2 mL HPLC crimp vials (Agilent Technologies, Waldbronn, Germany). The remaining 500 µL of supernatant was placed into the previously used spin filter, which was centrifuged again for 30 s at 2300× *g*. Filtrates of the same sample were combined and left to dry overnight in the laboratory fume hood. Dry samples were reconstituted in methanol to a defined volume of 500 µL. Then, the vial was crimped with a silicone septum (11 mm Silver Aluminum Crimp Cap, PTFE/silicone septa, Agilent Technologies, Waldbronn, Germany) and vortexed to ensure the complete solution of all sample constituents.

### 5.3. Analysis via Ultra-Performance Liquid Chromatography Coupled with Tandem Mass Spectrometry

**Figure 7:**
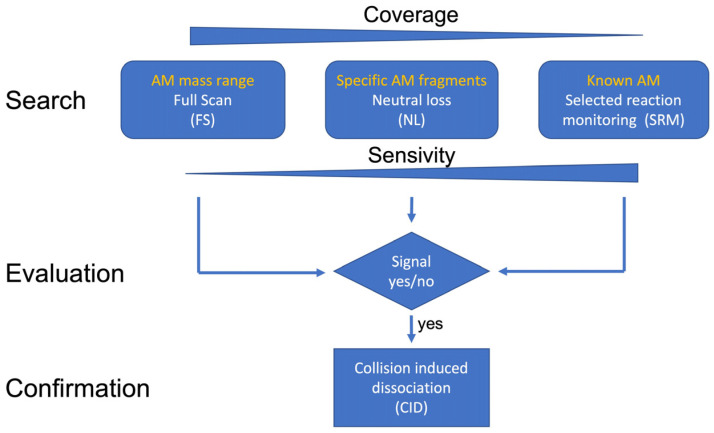
Conceptual model of the analytical approach of the detection of potential new AMs and flow chart of the applied MS/MS modes.

An ultra-performance liquid chromatography (UPLC^®^) instrument coupled with tandem mass spectrometry (MS/MS) was used to identify and quantify the toxin levels of AMs. The UPLC system included an ACQUITY UPLC column oven, an AQUITY UPLC I-class autosampler, and an ACQUITY UPLC I-class binary pump. The separation was created through a Purospher^®^ STAR RP-18 endcapped (2 µm) Hibar^®^ HR 50-2.1 UPLC column (Merck Millipore, Darmstadt, Germany). A 0.5 µm OPTS-SOLV^®^ EXP™ precolumn (Sigma-Aldrich, Hamburg, Germany) was used to ensure the safety of the column. The entire system was coupled to a Xevo^®^ TQ-XS mass spectrometer (Waters GmbH, Eschborn, Germany). The software MassLynx (Version 4.2, Waters, Eschborn, Germany) was used to collect and analyze the data. Detection limits were defined to be threefold the signal-to-noise ratio (S/N), which were also directly calculated in TargetLynx XS [17]. Table 7 lists the implemented chromatography and mass spectrometry parameters for the UPLC-MS/MS measurements. Figure 7 shows the conceptual model of the analytical approach used to detect potential new AMs and a flow chart of the applied MS/MS modes.

#### 5.3.1. Selected Reaction Monitoring (SRM) Mode

For the detection of known AMs and their quantification, the SRM mode was applied. Table 8 provides an overview of the transitions of every known AM, as well as for the AMs detected by Wellkamp et al. [17], named N1 to N16, and three new variants described in this paper (U1, U2, and U3).

The limits of detection (LoD) were calculated based on the signal-to-noise ratio (S/N) of the luteophanol-D (LPD) standard in ng µL^−1^. The LoD was expressed as fg sample^−1^. The concentration was calculated with Equation (1).

Equation (1):

(1) $$\mathrm{LoD}\left[\mathrm{fg}\;{\mathrm{cell}}^{-1}\right]=c\left(\mathrm{LPD}\right)\;*\;\frac{3}{\frac{S}{N}\left(\mathrm{LPD}\right)}\;*\;\mathrm{sample}\;\mathrm{volume}\left[µL\right]\;*\;\frac{1,000,000}{\mathrm{number}\;\mathrm{of}\;\mathrm{cells}}$$

where LoD = limit of detection; c(LPD) = concentration of the luteophanol-D standard in ng µL^−1^; and S/N (LPD) = signal-to-noise ratio of the luteophanol-D standard.

Identified AMs were calibrated against the LPD standard solution and expressed as LPD equivalents according to Equation (2). 

Equation (2):

(2) $$\mathrm{Toxin}\left[\mathrm{ng}\;{µL}^{-1}\right]=\frac{\mathrm{Peak}\;\mathrm{Area}\;\left(\mathrm{Toxin}\right)\;*\;c\left(\mathrm{LPD}\right)}{\mathrm{Peak}\;\mathrm{Area}\;\left(\mathrm{LPD}\right)}$$

where Toxin = concentration of measured toxin in ng µL^−1^; Peak Area (Toxin) = obtained peak area calculated by MassLynx from the toxin; c(LPD) = concentration of the luteophanol-D standard; and Peak Area (LPD) = obtained peak area calculated by MassLynx from the LPD standard.

AMs were calibrated against an external LPD standard and expressed as LPD equivalents. Equation (3) shows the calculation of the cell quota in fg cell^−1^.

Equation (3):

(3) $$\mathrm{Toxin}\;\mathrm{cell}\;\mathrm{quota}\;\left[\mathrm{fg}\;{\mathrm{cell}}^{-1}\right]=\frac{\mathrm{Toxin}\;[\mathrm{ng}\;{µL}^{-1}]\;*\;\mathrm{Sample}\;\mathrm{volume}\;\left[µL\right]\;*\;1,000,000}{\mathrm{Number}\;\mathrm{of}\;\mathrm{cells}}$$

where Toxin cell quota = concentration of toxin in fg per individual cell and Toxin = concentration of measured toxin in ng µL^−1^.

The LPD was isolated from the *Amphidinium carterae* strain ACRN03 [27]. Ten µg of the LPD was kindly provided by F. García-Camacho of the University of Almería, Spain. The powdery standard material was dissolved in 800 µL of methanol, resulting in an LDP concentration of about 13 ng µL^−1^. All quantitative values presented in this work are expressed as LPD equivalents and are therefore only semi-quantitative. 

#### 5.3.2. Neutral Loss (NL) Measurement Mode

To date, fourteen neutral AM fragments have been identified as a result of the characteristic C-1’/C-1 bond cleavage between the lipophilic and hydrophilic part of AMs. Seven of them are nonsulfated neutral fragments, whilst the other seven are sulfated neutral fragments (Table 1). All strains of this study were screened in the neutral loss (NL) mode for each of these 14 neutral losses.

Each strain was checked for peaks resulting from the above-mentioned NLs. A signal-to-noise ratio of three was set as the minimum threshold for the selection of peaks. The Q1-Masses of the detected peaks were further examined via the product ion spectra of the respective precursors. 

#### 5.3.3. Full-Scan (FS) Measurement Mode

The full-scan measurements in the mass range from *m*/*z* 1000 to 1800 were used in addition to NL scans to ensure that potential AM variants would be detected in case they did not form any of the known neutral losses listed in Table 1. Every peak that showed a retention time in the range of the known AMs, which is between 2.0 and 4.2 min, was checked for the presence of abundant Q1-Masses. 

#### 5.3.4. Collision-Induced Dissociation (CID) Measurement Mode

Customized collision-induced dissociation experiments were generated for all *m*/*z* values detected by the NL and FS experiments for further investigation of the discovery of possible new AM variations. The starting mass stayed consistently at *m*/*z* 100 for every generated CID scan. The end mass had a value of about *m*/*z* 100 more than the recorded Q1-Mass. Ten thousand (10,000) was the set scanning rate of the mass spectrometer and was therefore kept consistent. The collision energy was consistently at 75 eV. The time frame of the CID scan was based on the respective retention time. The start was selected to be one minute earlier than the retention time, whilst the end was set to be one minute after the stated retention time. A time window of 2 min was therefore used in every CID scan. The flow state into the liquid chromatography was set to start 0.01 min after the starting time of the measurement and was switched from the chromatography path into the waste path 0.01 min before the measurement window ended. 

#### 5.3.5. Selection of Neutral Loss Scans

**Figure 8:**
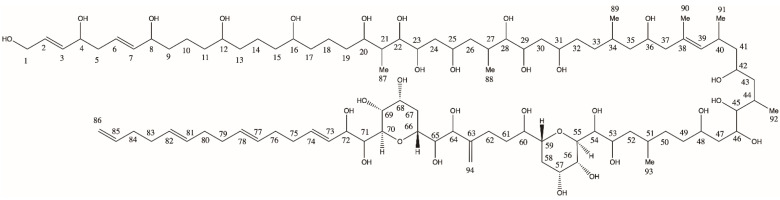
Original carbon numbering of AM21 by Satake et al. [16].

**Figure 9:**
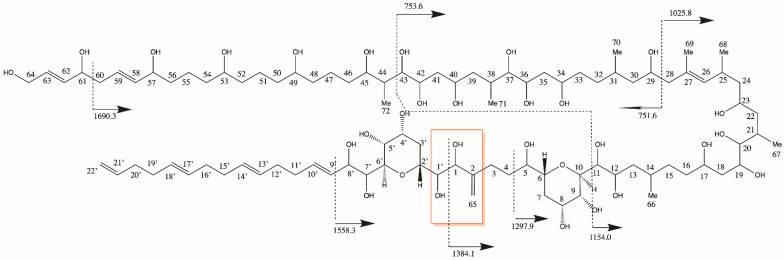
Fragmentation scheme of AM21 [17] and modified carbon numbering. The C-1′/C-1 bond cleavage site is marked in the red frame.

A common feature among all AMs is a bond cleavage between two vicinal hydroxyl groups near tetrahydropyran ring B in the conserved central part of the molecules, separating AMs into the hydrophilic and lipophilic parts. With carbon numbering starting at the end of the molecule according to the IUPAC (Figure 8), the C numbers at the conserved cleavage site would depend on the carbon number of the hydrophilic parts of the molecules, and thus, result in different numbers across different AMs. To identify and visualize similarities and differences between lipophilic and hydrophilic parts of AMs, a numbering starting at both sides of the cleavage site has been proposed, where C-1′ to C n’ denominates the lipophilic part and C-1 to C-n the hydrophilic part of AMs [17] (Figure 9).

Due to the high polarity of the hydrophilic part (C-1 to C-n), it usually carries the charge after ionization of the ion source and results in the charged fragment after the C-1/C-1′ cleavage, whereas the lipophilic part results in a neutral fragment. This neutral fragment includes the entire lipophilic part starting from the C-1′ atom. The lipophilic parts of AMs are relatively conserved among different AM variants and have only a few variations compared to the hydrophilic parts. To date, a total of seven pairs of variations of the lipophilic parts have been reported in the 37 described AMs from the literature. These pairs can be divided into nonsulfated and sulfated AMs (Table 1); therefore, the method parameters need to be slightly adjusted to allow for the successful detection of AMs. Some sulfated AMs lose their sulfate group before they are further fragmented [17]. The loss of a sulfate group occurs in the form of NaHSO_4_ (120 Da) from [M+Na]^+^. A total of 14 different neutral losses are currently known to exist in AMs. 

## Figures and Tables

**Table 1:** Observed nonsulfated and sulfated neutral losses from AMs, their *m*/*z* values, the number of C atoms, hydroxyl groups, and double bonds.

Number of Neutral Losses	Neutral Loss (*m*/*z*)	C Atoms	Hydroxyl Groups	Double Bonds
**Nonsulfated**				
1	392.13	22	5	5
2	426.23	22	7	4
3	418.24	24	5	6
4	398.28	22	5	2
5	440.25	23	7	4
6	442.23	22	8	4
7	338.18	18	5	4
**Sulfated**				
1.1	512.13	22	5	5
2.1	546.23	22	7	4
3.1	538.24	24	5	6
4.1	518.28	22	5	2
5.1	560.25	23	7	4
6.1	562.12	22	8	4
7.1	458.18	18	5	4

**Table 2:** Detected neutral losses (NLs) and corresponding Q1-Masses found in various strains.

Strain	*t_R_* (min)	Q1-Mass (*m*/*z*)	Neutral Loss
AxLT113	3.75	1114	518
AxLT111	2.93	1138	338
AxLT111	3.90	1140	392
AtLPZ38	2.03	1238	440
AxSQ175	2.90	1242	392/512
AxSQ175	3.80	1264	392
AA39	2.90	1296	426
AeSQ172	3.79	1305	442
AA60	3.75	1384	392
AxSQ175	3.90	1392	442
AxSQ175	3.90	1396	392
AA60	2.92	1418	426
AA60	2.85	1486	512
AeSQ172	3.77	1764	440

**Table 3:** Additional detected Q1-Masses through the full-scan measurement mode (FS).

Strain	$t_{R}$ (min)	Q1-Mass (*m*/*z*)
AxLT113	4.16	1028
AxLT113	4.16	1044
AxRoq139	3.73	1074
AxLT113	3.78	1134
AxSQ175	4.04	1290
AA60	3.03	1334

**Table 4:** Confirmed new AM variants, their retention times, Q-1 Masses, detection mode, and operational names.

Strain	$t_{R}$ (min)	Q1-Mass (*m*/*z*)	Detection Mode	Variant Name
AxSQ175	2.90	1242	Neutral loss	U1
AA60	3.75	1384	Neutral loss	U2
AA60	2.85	1486	Neutral loss	U3

**Table 5:** Quantification of known AMs from cultivated *Amphidinium* isolates from Mexico, reported in fg cell^−1^.

AM	$t_{R}$ (min)	Strain/Species						
		AA39 *A. massartii*	AA60 *A. operculatum*	AxLT111 *	AeSQ 172 *A. eilatiensis*	AxSQ175 *	AeSQ177 *A. eilatiensis*	AeSQ181 *A. eilatiensis*
AM02	3.78		4	21	200	22	231	632
AM04	3.87				441		547	2
AM05	4.07				317		406	1
AM06	3.18		22					
AM07	3.3				45		79	250
AM09	4.10				1		19	796
AM11	3.43				2		5	5
AM14	2.61		2				10	2
AM15	3.23	1						
AM17	4.17			2		5		2
N7	4.20							2
N8/N9	4.17							2
N12	3.88				14		15	
N13	3.52				1		2	
U1	2.90					985		
U2	3.75		32					
U3	2.85		79					
Total AM content		1	139	23	1021	1012	1314	1694

* Unidentified *Amphidinium* cf. *carterae* species.

**Table 6:** Isolate origin, date of sampling, and species.

Isolate	Locality of Origin *	Geographical Location	Date of Sampling	Species **
AtLPZ38	Balandra Bay, Baja California Sur, (1)	Gulf of California	January 2018	*A. theodorei*
AA39				*A. massartii*
AA40				*A. theodorei*
AA60	Veracruz Reef System, Veracruz (2)	Gulf of Mexico	May 2018	*A. operculatum*
AxLT111	Laguna de Términos, Campeche (3)		January 2019	Unidentified
AxLT112				*A. massartii*
AxLT113				Unidentified
AxRoq139	Roqueta Island, Acapulco, Guerrero (4)	Mexican South Pacific	June 2019	Unidentified
AeSQ172	San Quintín, Baja California (5)	Mexican North Pacific	December 2019	*A. eilatiensis*
AxSQ175				Unidentified
AeSQ177				*A. eilatiensis*
AeSQ181				*A. eilatiensis*

* (Numbers) indicate the sampling site in Figure 6. ** Species have been determined through morphological and molecular analysis (not shown), except for those designated as unidentified.

**Table 7:** Chromatographic and mass spectrometric parameters for the measurement of AMs for all three measurement modes (NL, FS, CID).

**Chromatographic Parameters**	
Eluent composition	A: 500 mL ultrapure water + 252.5 µL NH_4_OH (25%) B: 450 mL acetonitrile + 50 mL ultrapure water + 252.5 µL NH_4_OH (25%)
Eluent gradient	80% Eluent A to 20% Eluent B
Total duration (min)	5
Flow rate (mL min^−1^)	0.2
Injection volume (µL)	0.5
Collision energy (eV)	75 85 for AMs over 1500 *m*/*z* during product ion scans
Scanning time (sec)	0.133
Scanning rate (points per peak)	12
**Spectrometric Parameters**	
Ion source	
Capillary voltage (kV) Cone voltage (V) Desolvation temperature (°C)	3.00 40 600
Gas flow	
Desolvation gas flow (L h^−1^) Cone gas flow (L h^−1^) Nebulizer gas flow (bar)	1000 150 7.0
Further settings	
Autosampler temperature (°C) Column temperature (°C) Electrospray Full-scan mass range (*m*/*z*)	10 40 ES+ 1000–1800

**Table 8:** Overview of known AM transitions.

Toxin	Q1-Mass (*m*/*z*)	Q3-Mass (*m*/*z*)	Toxin	Q1-Mass (*m*/*z*)	Q3-Mass (*m*/*z*)
AM1	1512	974	LP-A	1278	754
AM2	1398	1006	LP-B	1344	904
AM3	1350	932	LP-C	1344	904
AM4	1324	932	LP-D	1330	904
AM5	1394	976	LS	1374	976
AM6	1368	976	LS-A	1296	904
AM7	1254	742	LS-B	1266	754
AM9	1350	932	SP	1266	754
AM10	1296	904	N1	1268	876
AM11	1500	988	N2	1430	1038
AM12	1426	914	N3	1458	1066
AM13	1452	914	N4	1316	876
AM14	1288	742	N5	1346	946
AM15	1186	760	N6	1346	946
AM17	1306	816	N7	1346	928
AM18	1382	964	N8	1364	946
AM19	1484	946	N9	1364	946
AM20 (M) *	1346	904	N10	1364	946
AM20 (S) **	1653	1260	N11	1364	946
AM21	1798	1406	N12	1326	946
AM22	1668	1330	N13	1326	928
AM-A	1362	964	N14	1344	946
AM-B	1464	946	N15	1344	946
AMD-G	1300	768	N16	1344	946
CAR-E	1422	1030	U1	1242	730
KAR-A	1480	1082	U2	1384	992
KAR-B	1462	1064	U3	1486	974

AM = amphidinol; AMD = amdigenol; CAR = carteraol; KAR = karatungiol; LP = luteophanol; LS = lingshuiol; SP = symbiopolyol; unknown AM variants named N1-N16 described by Wellkamp et al. [17]. The remaining unknown AM variants, named U1, U2, and U3, are described in this work. * Molina et al., 2018 [27]; ** Satake et al., 2017 [16].

## Data Availability

The data presented in this study are available in this article and supplementary material here.

## Supplementary Materials

The following supporting information can be downloaded at: https://www.mdpi.com/article/10.3390/toxins15020163/s1, Table S1: Relative AM, estimated as LPD equivalents.
