# Supplementary material for: First Identification of Amphidinols from Mexican Strains and New Analogs
Source: Toxins (Basel). 2023 Feb 16;15(2):163. doi: 10.3390/toxins15020163 (PMC9961859; doi:10.3390/toxins15020163)
Supplement: Supplementary file 1 [file toxins-15-00163-s001.zip › toxins-2107612-supplementary.pdf]

toxins

Table S1. Relative AM, estimated as LPD equivalents.

AM	Strain / Species						
	AA39 *A. massartii*	AA60 *A. operculatum*	AxLT111 **	AeSQ172 *A. eilatiensis*	AxSQ175 **	AeSQ177 *A. eilatiensis*	AeSQ181 *A. eilatiensis*
AM02		2.9	91.3	19.6	2.2	17.6	37.3
AM04				43.2		41.6	0.1
AM05				31.0		30.9	0.1
AM06		15.8					
AM07				4.4		6.0	14.8
AM09				0.1		1.4	47.0
AM11				0.2		0.4	0.3
AM14		1.4				0.8	0.1
AM15	100.0						
AM17			8.7		0.5		0.1
N7							0.1
N8/N9							0.1
N12				1.4		1.1	
N13				0.1		0.2	
U1					97.3		
U2		23.0					
U3		56.8					
**% total**	**100.0**	**100.0**	**100.0**	**100.0**	**100.0**	**100.0**	**100.0**

** = unidentified species.
